# Infectious diseases linked to cross-contamination of flexible endoscopes

**DOI:** 10.1186/2047-2994-4-S1-P57

**Published:** 2015-06-16

**Authors:** N Kenters, EGW Huijskens, C Meier, A Voss

**Affiliations:** 1 Infection Prevention and Control, Albert Schweitzer Hospital, Dordrecht, Netherlands; 2 Medical Microbiology, Albert Schweitzer Hospital, Dordrecht, Netherlands; 3 Infection Prevention and Control, Rivierenland Hospital, Tiel, Netherlands; 4 Medical Microbiology and Infectious Diseases , Radboud University Medical Centre, Nijmegen, Netherlands

## Introduction

Flexible endoscopes are widely used to examine, diagnose and treat medical disorders. Despite the availability of international, national and local endoscope reprocessing guidelines, contamination and transmission of microorganisms continue to occur.

## Objectives

This article presents an overview of publications in case reports and outbreaks related to contamination of flexible endoscopes.

## Methods

The following search terms or combinations of terms were used to search in PubMed: *endoscope*, *endoscope reprocessing*, *outbreak* and *infection.* Studies were included if published in English from the year 2000 onwards.

## Results

Thirty-two publications were included in this review. From these, eight incidents involved damaged or defective flexible endoscopes, eight were related to failures during manual endoscope reprocessing, eleven reports related to reprocessing failures where the disinfection step was carried out by an AER and five to due failure or malfunctioning of the AER.

## Conclusion

To ensure quality reprocessing of flexible endoscopes, mandatory competency training and periodic auditing should take place. Early detection of contamination would be made easier if periodic microbiological testing were to be included in the guidelines. The guidelines need to include a standardized procedure, to ensure maximum effectiveness. AERs are often used for flexible endoscope reprocessing and therefore should be included in the guidelines. Periodic maintenance on flexible endoscopes and AERs should always be carried out as the manufacture advises. Mandatory reporting of lapses will give a better overview of cross-contamination of flexible endoscopes worldwide.

## Disclosure of interest

None declared.

